# Erg Channel Is Critical in Controlling Cell Volume during Cell Cycle in Embryonic Stem Cells

**DOI:** 10.1371/journal.pone.0072409

**Published:** 2013-08-02

**Authors:** Shaimaa Abdelhady, Satish Srinivas Kitambi, Vanessa Lundin, Roland Aufschnaiter, Petra Sekyrova, Indranil Sinha, Kalle T. Lundgren, Goncalo Castelo-Branco, Sten Linnarsson, Roland Wedlich-Söldner, Ana Teixeira, Michael Andäng

**Affiliations:** 1 Department of Physiology and Pharmacology, Karolinska Institutet, Stockholm, Sweden; 2 Department of Medical Biochemistry and Biophysics, Karolinska Institutet, Stockholm, Sweden; 3 Department of Cell and Molecular Biology, Karolinska Institutet, Stockholm, Sweden; 4 Cellular Dynamics and Cell Patterning, Max-Planck Institute of Biochemistry, Martinsried, Germany; 5 Department of Reconstructive Plastic Surgery, Karolinska University Hospital, Stockholm, Sweden; Baylor College of Medicine, United States of America

## Abstract

The cell cycle progression in mouse embryonic stem cells (mESCs) is controlled by ion fluxes that alter cell volume [1]. This suggests that ion fluxes might control dynamic changes in morphology over the cell cycle, such as rounding up of the cell at mitosis. However, specific channels regulating such dynamic changes and the possible interactions with actomyosin complex have not been clearly identified. Following RNAseq transcriptome analysis of cell cycle sorted mESCs, we found that expression of the K^+^ ion channel Erg1 peaked in G1 cell cycle phase, which was confirmed by immunostaining. Inhibition of Erg channel activity caused loss of G1 phase cells via non-apoptotic cell death. Cells first lost the ability of membrane blebbing, a typical feature of cultured embryonic stem cells. Continued Erg inhibition further increased cell volume and the cell eventually ruptured. In addition, atomic force measurements on live cells revealed a decreased cortical stiffness after treatment, suggesting alterations in actomyosin organization. When the intracellular osmotic pressure was experimentally decreased by hypertonic solution or block of K^+^ ion import via the Na, K-ATPase, cell viability was restored and cells acquired normal volume and blebbing activity. Our results suggest that Erg channels have a critical function in K^+^ ion homeostasis of mESCs over the cell cycle, and that cell death following Erg inhibition is a consequence of the inability to regulate cell volume.

## Introduction

Ion channel activity has been shown to simultaneously affect cell cycle and cell volume in the S phase of the cell cycle in embryonic stem cells (ESCs) [1] potentially linking proliferation to physical behavior. ESCs have a characteristic round morphology throughout the cell cycle and they further round up at the onset of mitosis (Figure S1A,B). In contrast to ESCs, cells with a more flattened morphology, for example fibroblasts, round up exclusively at mitosis [2]. These morphology changes result from a balance between outward osmotic pressure versus an inward pressure generated by actomyosin contraction. Although regulation of actomyosin contractility during cell shape changes is relatively well understood [3], less is known about the repertoire of ion channels, transporters and pumps that may generate and regulate osmotic pressure during cell growth and division.

In osmotically challenged cells such as kidney cells, osmotic sensors act via volume regulatory ion transporters to re-establish osmotic homeostasis and maintain constant volume. During the tightly controlled processes of regulatory volume increase (RVI) and regulatory volume decrease (RVD) several classes of ion channels and transporters are coordinated to restore optimal cell volume. Na^+^/H^+^ exchangers, anion exchangers and Na^+^/K^+^/Cl^-^ co-transporters become active during RVI, while K^+^ channels, volume regulated anion channels and K^+^/Cl^-^ co-transporters are activated during RVD [4].

Activities of many transporters vary over the cell cycle. In particular, K^+^ channel activity controls progression from G1 to S phase [5] and is up regulated in rapidly proliferating cancer cells [6]. However, how exactly K^+^ flux regulates cell cycle progression is still not resolved. One potential downstream mechanism is the DNA damage response (DDR) pathway that can reversibly arrest ESCs in S-phase [1]. Similar to cancer cells, K^+^ channels control cell proliferation in mouse and human ESCs [7]. Here, we investigated K^+^ channel expression and activity in mouse ESCs (mESCs) during the cell cycle. We identified switches in K^+^ channel expression and a critical function for Erg K^+^ channel activity in maintaining volume homeostasis. Atomic force measurements revealed decreased cortical stiffness during small molecule inhibition of Erg channels, indicating an altered actomyosin organization in addition to an osmotic pressure increase. Decreasing intracellular osmotic pressure or blocking influx of K^+^ ions rescued cell viability and restored normal cell volume and blebbing [8] activity.

## Results

### Cell cycle regulated K^+^ channel expression

**Figure 1:**
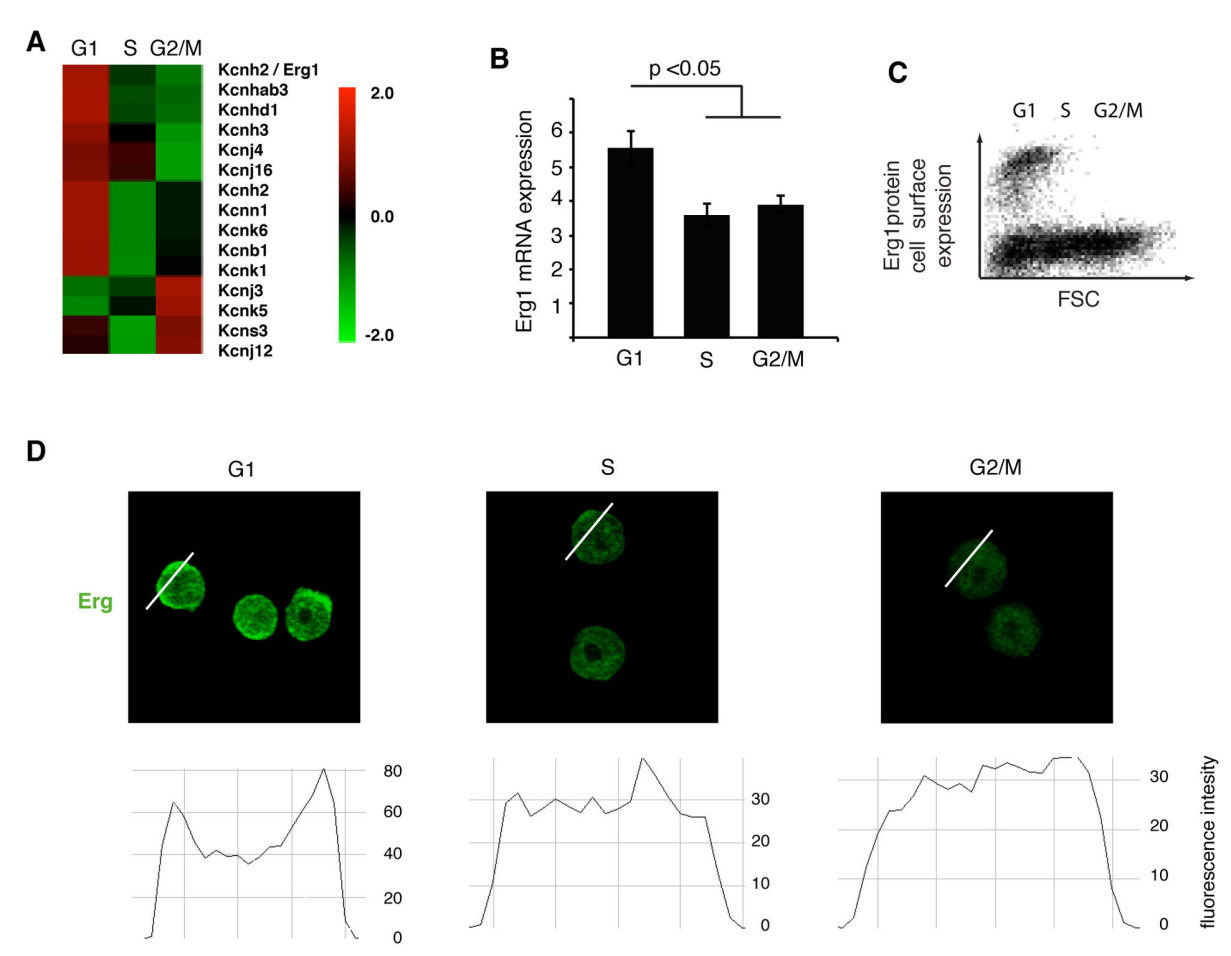
Cell cycle dependent Erg1 channel expression. (A) Heat-map displaying mRNA differential expression of selected K^+^ channels in different cell cycle phases (ANOVA-test p<0.005). (B) Erg1 mRNA expression level evaluated using real-time PCR in cell cycle sorted mESCs (ANOVA-test). (C) Flow cytometry plot of live mESCs stained for an extracellular epitope of Erg1 channel. (D) Confocal images of mESCs sorted in different cell cycle stages and immunostained for Erg1 protein with cross section histograms (note that measurements across nucleoli were avoided) showing Erg1 immunostaining intensity with increased plasma membrane localization in G1.

To identify channels with a cell cycle phase specific expression, we analyzed the mRNA transcriptome in mESCs by RNA sequencing after sorting G1, S and G2/M cell cycle phases using fluorescent flow cytometry. Several K^+^ ion channels exhibited cell cycle regulated expression. The highly expressed K^+^ channels Kcnc3 (Kv3.3) and Kcnh2 (Kv11.1, Erg1) had higher mRNA levels in G1 phase, while Kcnk5 (Task2), Kcns3 (Kv9.3) and Kcnj3 (Kir3.1) were mostly expressed in G2/M (Figure 1A, Figure S2). No K^+^ channels were selectively expressed during S phase. These data reveal a shift in K^+^ channel repertoire at the G1 -S and S - G2/M transitions.

Among the identified channels, Erg1, was particularly interesting since Erg channels have been shown to regulate cell proliferation [9] and development [10] and to play a role in cell volume regulation during osmotic challenge [11]. We therefore further analyzed expression of the Erg1 channel. Quantitative real-time RT-PCR of cell cycle sorted cells confirmed preferential expression in G1 (Figure 1B). To establish whether cell cycle specific Erg1 transcription correlated with protein expression levels, we performed live cell flow cytometry using a specific antibody targeting an extracellular epitope of Erg1. mESC size increased evenly over the cell cycle, with the smallest cells in G1 (Figure S3) and plotting forward scatter (indicating cell size) against Erg1 immunostaining showed that only the small G1 cells express Erg1 at the cell surface (Figure 1C). To further validate protein expression on a single cell level we cell cycle sorted ESCs and analyzed Erg1 expression by confocal microscopy. Erg1 protein preferentially localized into the plasma membrane with the highest intensity in G1 phase compared to S and G2/M (Figure 1D) indicating a dynamic Erg1 expression and possibly also channel activity peaking in G1.

### Erg inhibition causes cell death in G1 cell cycle phase independent of apoptosis

**Figure 2:**
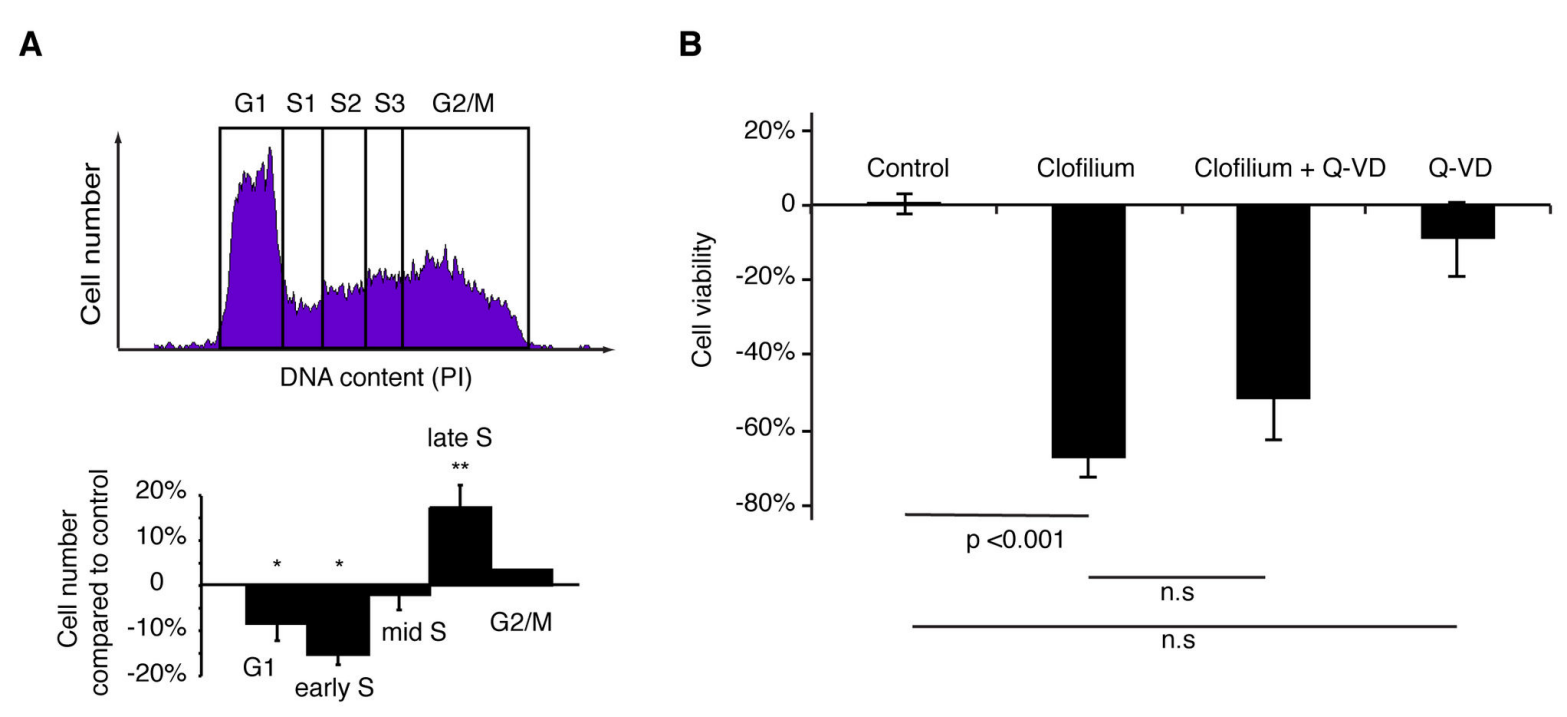
Inhibition of Erg activity results in a mainly apoptosis independent cell death in G1 and early S phase. (A) mESCs were exposed to cisapride (10 µM) or vehicle for 6 h and DNA content was assayed using propidium iodide labeling by flow cytometry and quantified in respective cell cycle stages (one-way ANOVA, * p<0.05, ** p<0.01). (B) mESCs were treated with the Erg inhibitor, E4031 (10 µM), for 24 h with and without apoptosis inhibitor, Q-VD-OPh (20 µM) and viability was measured using an ATP detecting viability assay. Data presented as mean ± SEM (N=3), one-way ANOVA, Tukey post-hoc test.

K^+^ channels control critical changes in membrane potential during the cell cycle [5]. As Erg1 was expressed on the cell surface in the G1 cell cycle phase, we next analyzed cell cycle profiles of cells exposed to Erg inhibitor clofilium (10 µM for 6 hours) using flow cytometry. We found a relative reduction in cell numbers in G1 and early S phase compared to late S or G2/M (Figure 2A). These results suggested that cells were indeed preferentially undergoing cell death in early cell cycle stage i.e. G1.

It has been shown that Erg channels play a role in apoptosis induced pathways via interaction with integrin and FAK signaling [9]. To investigate whether Erg inhibition resulted in cell death through apoptosis, we measured levels of cleaved caspase-3 by flow cytometry in mESCs exposed to clofilium (10 µM for 6 hours). We found that a subpopulation of cells had an increased level of cleaved caspase-3 hence undergoing apoptosis (data not shown). To assess the level of contribution of apoptosis to cell death induced by Erg inhibition, apoptosis was inhibited by co-administrating an irreversible inhibitor of caspase-3 activity (Q-VD-OPh, 20 µM), simultaneously with clofilium (10 µM). Surprisingly, we found that inhibition of apoptosis only slightly reduced cell death after Erg inhibition as measured by viability assay (Figure 2B). Thus, the majority of cells exposed to Erg inhibitor died via cell death mechanism other than apoptosis.

As we previously found that ion fluxes may activate the DDR pathway without apparent activation of apoptosis in ESC [1] we next analyzed the DDR pathway activation by immunostaining for Ser-139 phosphorylated histone H2AX (γH2AX). We found hyper-induced γH2AX levels in cellular fragments (Figure S4), which likely reflects genome-wide activation of DDR resulting from a large number of DNA damage sites caused by severe stress. Such high phosphorylation levels would not be expected during apoptosis as upregulated phosphatase activity normally accompanies the apoptotic program. This data therefore led us to suggest a non-apoptotic cell fragmentation process where the DDR pathway is at least partially functional.

### Erg inhibitor induced cell swelling and abolished blebbing

**Figure 3:**
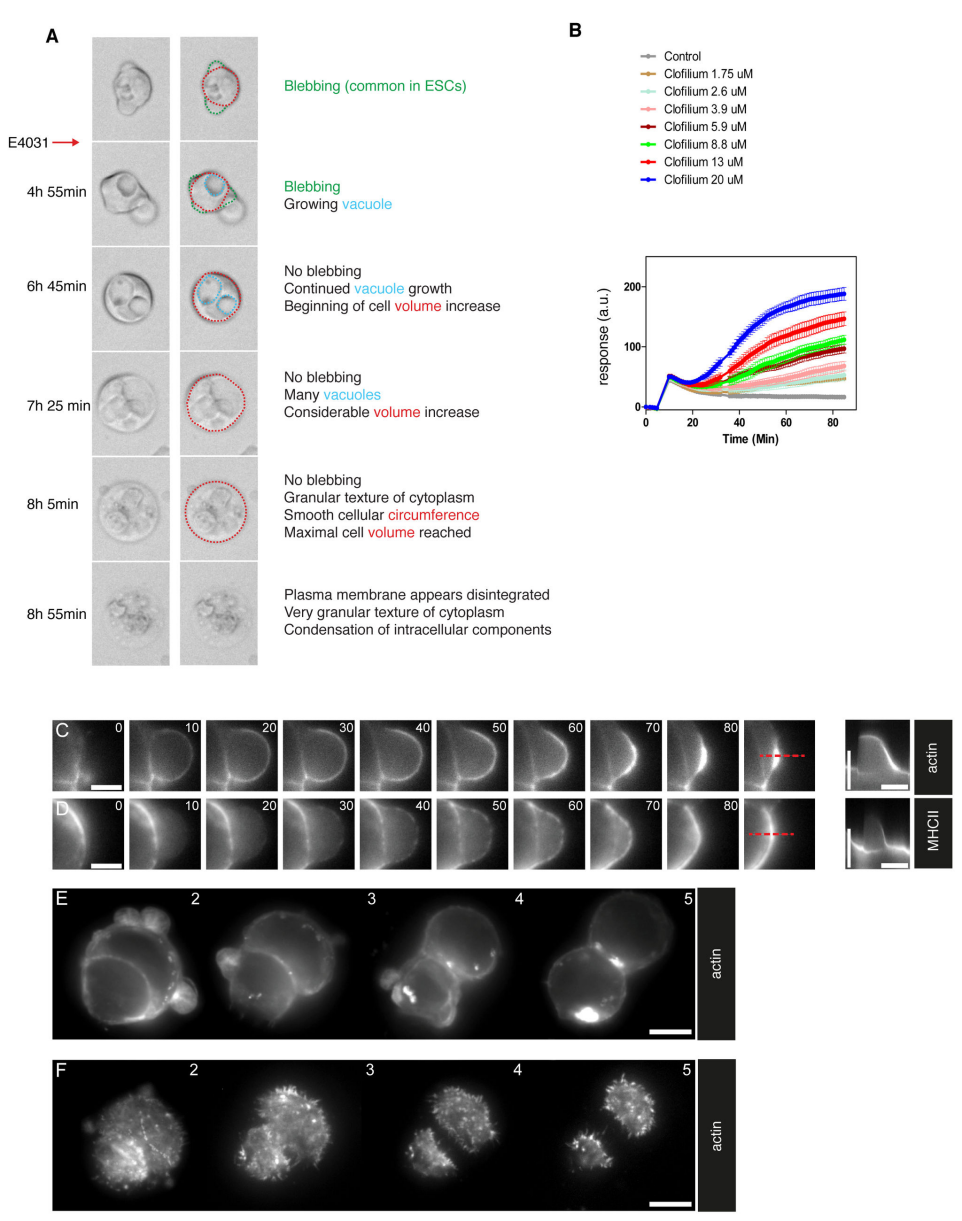
Inhibition of Erg results in cell volume increase and rupture. (A) Images from time-lapse movies of mESCs treated with Erg inhibitor, E4031 (10 µM). (B) Mass redistribution measurements of mESCs treated with different concentrations of clofilium. (C, D) Time-lapse images of fluorescent reporter for actin (Lifeact-mCherry) (C) and myosin MHCII (MHCIIA-GFP) (D) in a bleb. Kymographs (far right) of a bleb showing actin and myosin intensity over time. (E, F) Time-lapse images of actin (Lifeact-mCherry) during Erg inhibition by fluorescent microscopy (E) and TIRF microscopy (F). Scale bars in C and D are 4 µm and 10 µm in E and F. Time bars in the kymographs (C/D, vertical) are 100 s. Time stamps in C/D are in seconds and in E/F in hours.

To identify potential effects on cell behavior and morphology by Erg channel activity, we administered a selective Erg inhibitor E4031 (10 µM) during time-lapse recordings. In single cells, Erg inhibition abolished widespread spontaneous blebbing (Figure 3A) that was observed under control conditions (Figure S1A). Instead, cells increased in volume and after a stationary phase the plasma membrane rapidly expanded and ruptured, which was followed by aggregation of intracellular material (Figure 3A). This suggested that Erg activity is involved in maintaining volume homeostasis in mESCs. However, not all cells responded simultaneously, indicating a cell cycle stage correlated response. We observed that not until after 25 hours all cells appeared dead, which indicated that some cells might be able to pass at least one cell cycle under Erg inhibition.

To measure temporal kinetics of Erg inhibition as well as dose response we used an EPIC resonant wave guide grating biosensor, which monitors live cell relative mass redistribution (RMD). Cells reacted already 20 minutes after administration of a dose series of Erg inhibitor (clofilium) beginning at 1.75 µM (Figure 3B) suggesting early changes in morphology caused by Erg inhibition.

### Cortical actin dynamics attenuates after Erg inhibition

The large increase in cell volume indicated loss of control of cell morphology in which cortical actomyosin plays a major role. To directly monitor effects of Erg inhibition on actomyosin organization, we used fluorescent reporters and followed cell behavior in real time by time-lapse microscopy. Cortical F-actin was imaged after transfection with a Lifeact-mCherry expression construct and myosin with an MHCIIA-GFP construct [12] As expected under control conditions, blebbing in mESCs was accompanied by rapid accumulation of actin under the plasma membrane surface (Figure 3C). In contrast, myosin was recruited with a 20 second delay and was concentrated in distinct foci on the bleb surface (Figure 3D). Both, actin and myosin became concentrated during bleb retraction (Figure 3C, D).

After Erg inhibition, time lapse imaging of actin (Lifeact-mCherry) confirmed the loss of blebbing (Figure 3E). Imaging by total internal reflection fluorescence (TIRF) microscopy of Lifeact-mCherry revealed a reduction in the area of cell-surface contact upon Erg inhibition, reflecting the above described rounding up of cells (Figure 3F). Importantly, the density of cortical actin was not markedly affected, indicating that Erg channel activity did not play a major role in actin nucleation or organization (Figure 3F).

### Decreased cortical stiffness during Erg inhibition

**Figure 4:**
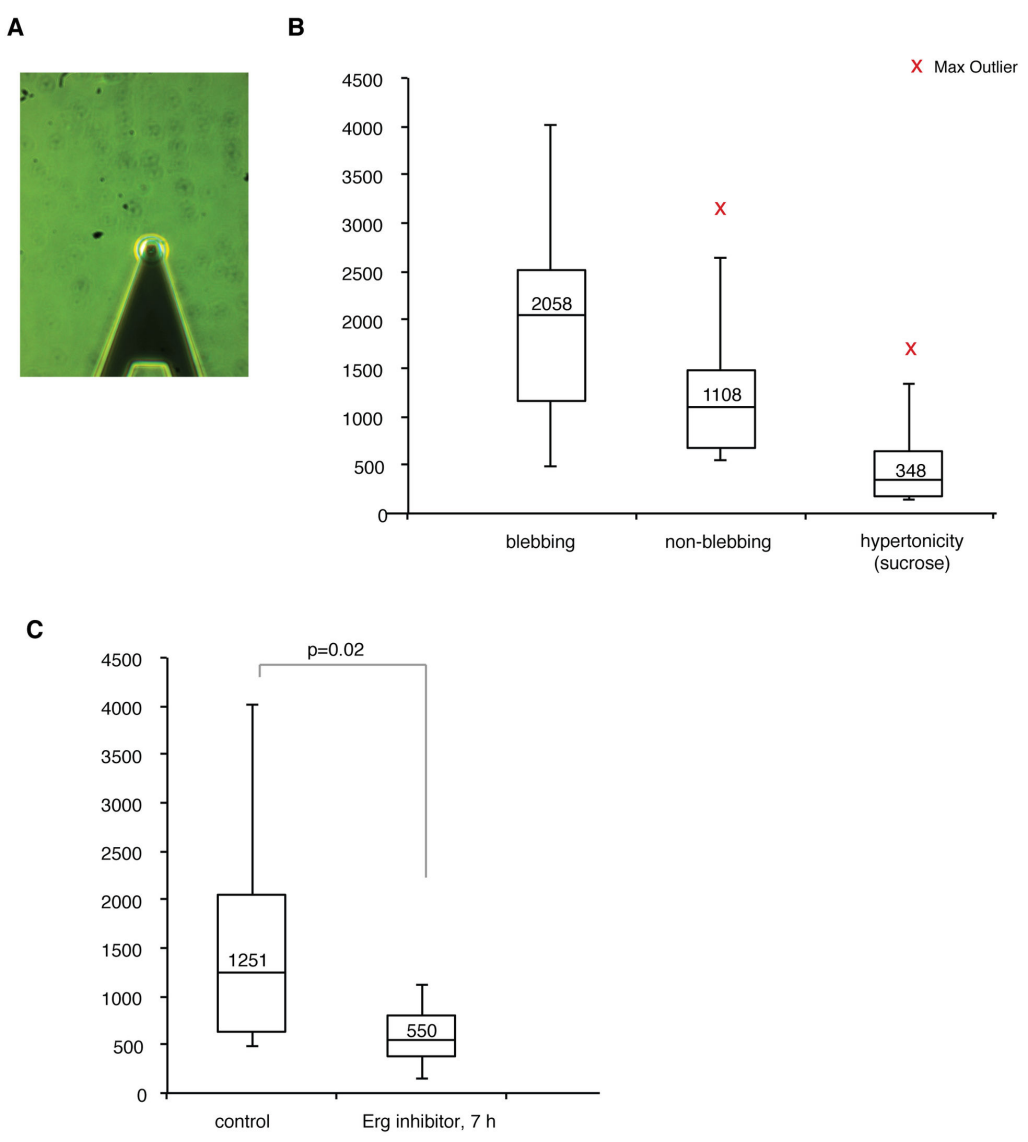
Erg inhibition decreased stiffness in mESCs. (A) A representative image of the cantilever placed over a mESC during atomic force microscopy. (B) In control conditions, blebbing cells showed a trend towards higher stiffness than non-blebbing cells (n=7, p=0.17, median indicated in box) and control cells that were subjected for hypertonic medium (sucrose 20 mM) for one hour showed reduced stiffness (n=11, p<0.001). (C) After 7 h of Erg inhibition (E4031; 10 µM) treated cells (n=8) were significantly (p=0.02, t-test unequal variance) less stiff than control cells (n=18).

The dramatic cell volume increase and the loss of blebbing during Erg inhibition indicated a reduction in cortical stiffness, which is critical to counteract osmotic pressure. Loss of actomyosin contractility would potentially allow a volume increase such as that observed during Erg inhibition. To address this directly, we measured cell stiffness after Erg inhibition using atomic force microscopy (Figure 4A). In control cells we found that under decreased osmotic pressure (hypertonic medium) cortical stiffness was reduced (Figure 4B). On the other hand we found cortical stiffness to be increased in blebbing vs. non-blebbing cells (Figure 4B). These results were consistent with adaptation in cortex organization and equilibrium between osmotic pressure and actomyosin contractility. After 7 hours of Erg inhibition we found a significant decrease in cell stiffness (2.5-fold, p=0.02, Figure 4C). This was in accordance with the time course for cell volume increase monitored by bright-field and fluorescent actin time-lapse imaging. Taken together, our data suggest that Erg inhibition caused a reduction of cortical stiffness, possibly through an effect on actomyosin contractility. This would then lead to an inability to withstand a gradually increased osmotic pressure.

### Erg-induced cell death acts via osmotic and K^+^ ion homeostasis

**Figure 5:**
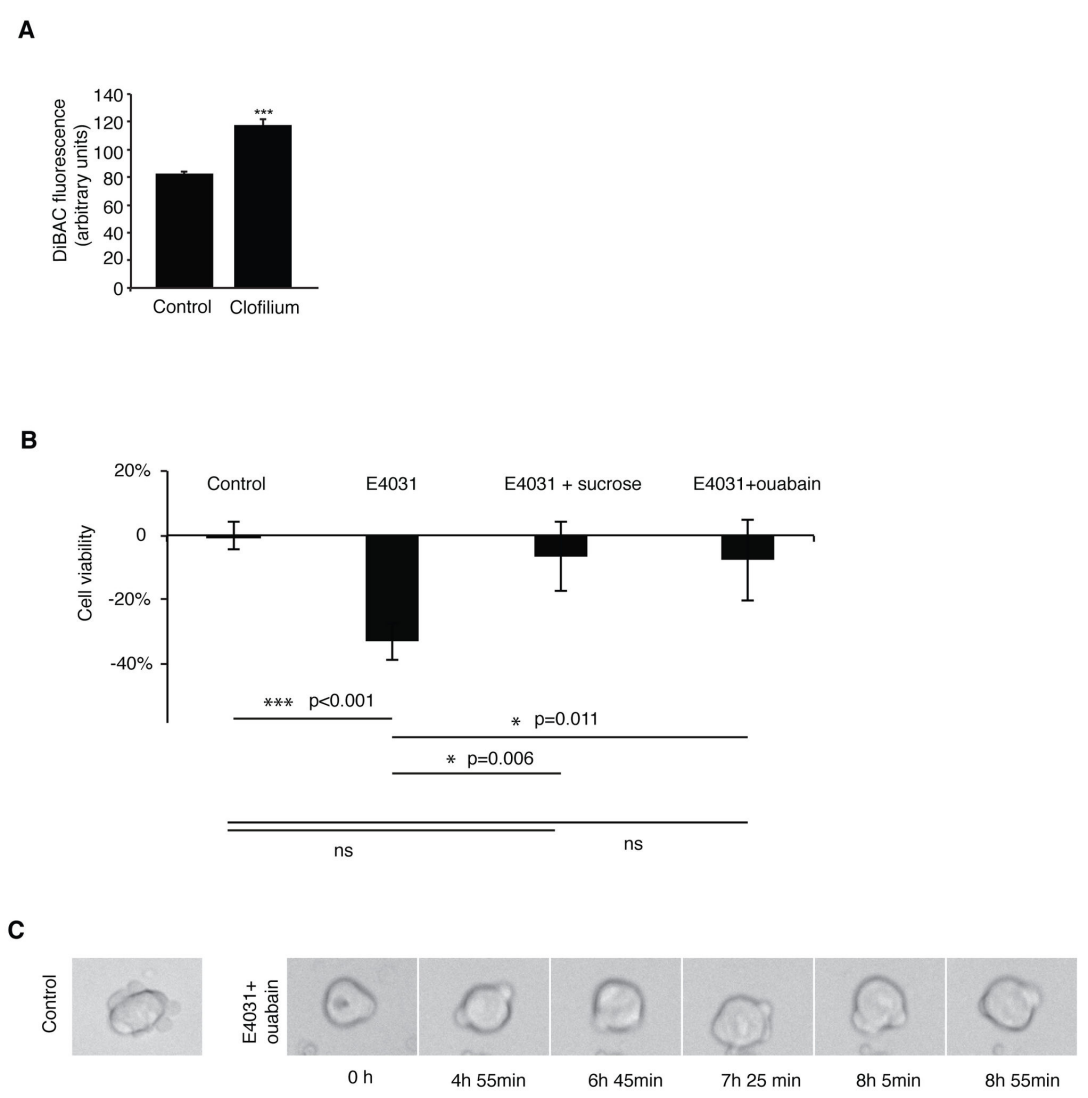
(A) Flow cytometric recordings of DiBAC_4_(3) loaded mESCs (n=10 000 cells, N=3) treated with 10 µM clofilium for 1 h. (B) Cell viability of mESCs treated with E4031 with and without sucrose (20 mM) or ouabain (1 µM) for 24 h. Data presented as mean ± SEM (n=4), one-way ANOVA, Tukey’ post-hoc test. (C) Images from time-lapse movies of mESCs treated with Erg inhibitor, E4031 (10 µM) and with the Na^+^,K^+^-ATPase inhibitor ouabain (1 µM).

To evaluate net effects on ion fluxes by Erg inhibition, we next analyzed membrane potential 1 hour after compound addition (clofilium, 10 µM) using a membrane potential sensitive reporter dye (DiBAC) and live cell flow cytometry. We found that membrane potential over the plasma membrane decreased as shown by the increase in fluorescence intensity (Figure 5A), in accordance with the expected accumulation of K^+^ ions in the cell when inhibiting Erg channels. As Erg inhibition caused plasma membrane depolarization, we hypothesized that inhibition of Erg activity caused accumulation of intracellular K^+^ ions leading to a changed equilibrium between osmotic pressure and the cortical actomyosin eventually resulting in cell rupture. To test this idea, various conditions that would counteract increase of intracellular osmolytes were applied. First, Erg inhibitors were co-administered with sucrose to test whether hyper-osmolarity could restore cell viability. Indeed, moderate increases in osmolarity (20 mM sucrose) rescued viability of mESCs after 24 hours (Figure 5B).

Osmotic homeostasis is maintained by the sodium pump (Na, K-ATPase) which exports 3 Na^+^ ions out of the cell while importing 2 K^+^ ions inside the cell. K^+^ ions are subsequently exported via K^+^ channels. This net export of ions accommodates import of osmolytes such as amino acids, glucose and other small molecules in the cell. We argued that Erg channels might interact with the sodium pump in this K^+^ circuit and cause K^+^ over-accumulation when inhibited. To test this, inward flow of K^+^ ions was blocked using ouabain (1 µM), a selective inhibitor of Na, K-ATPase. Co-administration of Erg inhibitor and ouabain increased cell survival after 24 hours (Figure 5C) which suggests that, Na, K-ATPase functionally interacts with Erg channels to control intracellular K^+^ and volume homeostasis.

## Discussion

**Figure 6:**
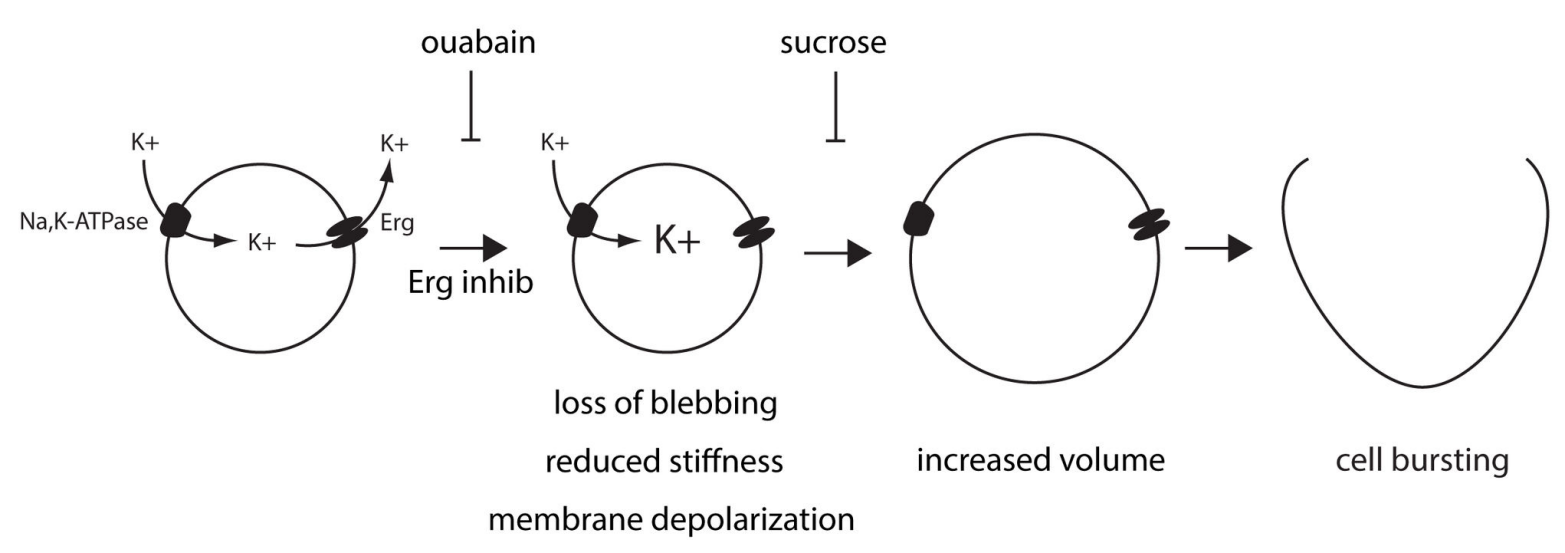
K^+^ permeability via Erg channel activity is critical for cell volume homeostasis. The Na, K-ATPase and K^+^ channels cooperate to establish a K^+^ ion circuit controlling intracellular [K^+^]. Inhibition of Erg channels lead to altered equilibrium between osmotic pressure and cortical actomyosin function resulting in an increase in cell volume ending in cell bursting. This could be counteracted by increasing extracellular osmolarity with sucrose or by blocking the influx of K^+^ ions by inhibiting the Na, K-ATPase with ouabain.

Erg channels have previously been shown to be involved in cell cycle progression as well as in maintenance of cell volume during osmotic stress [11]. Here we demonstrated that Erg channels play a role in regulating ion homeostasis and cell volume under iso-osmotic conditions (Figure 6). Mitotic cells exhibit an increased rounding force [2] and in the G1 phase, osmotic pressure and cell morphology are restored to a more relaxed state. The finding that Erg channels have a high cell surface expression in G1, suggests that Erg channels may act to modulate the rounding forces after mitosis. Increased cell volume during Erg inhibition may be a consequence of an increase in intracellular K^+^ ions leading to either (1) increased intracellular osmotic pressure or (2) a more direct effect on cortical actomyosin function as suggested by the reduced stiffness measured by atomic force microscopy. In another cell type, endothelial cells, cortical stiffness has been reported to be similarly controlled by changes in ion fluxes including increased extracellular K^+^ levels [13,14].

Erg inhibition most likely resulted in increased intracellular K^+^ observed as membrane depolarization in our experiments. Judging from the reported investigations in endothelial cells, either cations (in particular K^+^) or depolarization may cause reduced stiffness and increased cell volume [13,14]. It is difficult to experimentally dissociate these functions, but our data from inhibition of the Na, K-ATPase indicate a resolving strategy. As the Na, K-ATPase exports three and imports only two cations the net result is membrane hyperpolarization. Block of the Na, K-ATPase may thus not counteract depolarization, such as we found during Erg inhibition. Nonetheless, such block counteracted volume effects elicited by Erg inhibition, and our data therefore do not support that depolarization per se may drive the observed cell volume increase. Most likely the observed effects on cell volume in ESCs therefore were a consequence of increased K^+^ ion concentration.

Erg channels play a role in cell-cell contacts and cell motility via interactions with integrins, FAK (focal adhesion kinase) and Rac1 [15,16]. It is therefore possible that swelling induced disruption of structural integrity of the plasma membrane and the cytoskeleton during Erg inhibition in mESCs has a strong impact on several signaling pathways downstream of membrane bound receptors, such as the complexes formed between Erg channels and β1-integrins. In such a case Erg function would be positioned upstream of integrin/FAK/myosin signaling. The data that Erg controls cell volume and thus cell shape and cell-cell contacts, confirm that Erg function may be critical in this upstream position. Although lack of integrin signaling is unlikely to play a fundamental role in mESC survival on a shorter time scale, as these cells can readily be passaged as single cells, it may have importance in other cells types. For example, human ESCs when dissociated undergo cell death accompanied by intense plasma membrane blebbing via hyper activated actomyosin [17,18]. This sensitivity is shared with pluripotent mouse epi-stem cells (EpiSCs) derived from the post-implantation epiblast [18]. The identity of the dissociation-associated stimulus that triggers hyper activation of actomyosin in human ESCs and mouse EpiSCs remains unknown. Interestingly, Erg expression was found to be absent in human ESCs [19], which may be explained by their relatively later developmental identity as EpiSCs [20]. As Erg inhibition caused relaxation of the actomyosin in mouse ESC, it could be interesting to study whether the lack of ERG expression in human ESCs is at least a partial explanation for the sensitivity to single cell passaging. The commonly used mechanical separation of larger cell aggregates during passaging of human ESCs may maintain buffering of osmotic pressure via e.g. gap junctions circumventing requirement of ERG activity.

In conclusion, in mouse ESCs, Erg1 expression is transcriptionally hardwired by the cell cycle and Erg channel activity is critical for controlling membrane potential, activity of cortical actomyosin and cell volume. Erg channels are thus an integral part of morphological changes accompanying the cell cycle.

## Methods

### Cell culture

R1 mESCs (kindly provided by A. Nagy, Samuel Lunenfeld Research Institute Mount Sinai Hospital [21]) were cultured in DMEM/F12 supplemented with N2 supplement, 0.4 mM 2- mercaptoethanol, 5 mM HEPES (all from Invitrogen), 10 ng/ml basic fibroblast growth factor and 1,000 U/ml ESGRO (Chemicon) in suspension as previously described [1]. For experiments, mESCs were grown on 0.2% gelatin coated six-well plates. Stock solution of doxorubicin was made by dissolving in distilled water to 1 mM, E- 4031 in distilled water to 10 mM and other Erg inhibitors in PBS/10% DMSO at 10 mM (all from Sigma).

### Cell sorting

mESCs were stained with 10 µg/ml Hoechst 33342 (Invitrogen) in DMEM/F12 medium supplemented with ESGRO and basic fibroblast growth factors at 37° for 30 min. Cells were sorted according to G1, S and G2/M cell cycle phases directly into RLT buffer (Qiagen) using Becton Dickinson FACSVantage/DiVa fluorescent cell sorter.

### RNA sequencing and data analysis

Total RNA was isolated from cell cycle sorted mESCs using RNeasy extraction mini kit (Qiagen). cDNA and sample preparation for RNA sequencing was done according to protocol (TruSeq RNA kit, Illumina). Samples were sequenced on an Illumina HiSeq 2000 sequencer as single-end 51-nucleotide reads according to the manufactures protocol. Raw reads were mapped to the reference mouse genome and normalized data was generated for each genomic feature using STRT software [22] Briefly, raw reads were aligned using Bow tie [23]. Mapped reads were normalized using RPKM (reads per KB million reads) normalization method [24]. Statistical analysis was done using Qlucore software.

### Real-time RT–PCR

A detailed description is given in [1]. In brief, total RNA was isolated with an RNeasy extraction mini kit (Qiagen), reverse transcription was performed with High Capacity cDNA Reverse Transcription Kit (Applied Biosystems), primers were designed with Primer Express software (Applied Biosystems) and real- time PCR was conducted with the SYBR green detection method on an ABI PRISM 7900 instrument (Applied Biosystems). Samples were run three times in triplicates.

Primer sequences

Erg1 Fw: CAG CCC GGG TCG ACAG

Erg1 Rev: CCC GGC CTG AAG CTG

### Immunostaining

Cell cycle sorted mESCs were plated on 0.2% gelatin-coated cover slips. Cells were fixed with 2% formaldehyde and permeabilized with 0.3% Tween for 10 min. Cells were incubated in PBS containing 1% BSA with following primary antibodies: Erg extracellular epitope (1:100 dilution, Almone Lab), Erg intracellular epitope (1:100 dilution, Abcam), Integrin α6 antibody (1:100 dilution, Millipore NAB1982). The nucleus was stained with DAPI and DAKO mouting media was used for mounting on slides. Olympus FluoView FV1000 confocal laser scanning microscope was used for acquiring and analyzing images.

### Cell viability assay

mESCs were plated as single cells in a 96-well plate and treated with Erg inhibitors for a period of 24 h. Cell viability was measured using CellTiter-Glo® Luminescent Cell Viability Assay (Promega), according to manufacturer’s instructions. Q-VD-OPh (R&D Systems) was dissolved in DMSO.

### DiBAC staining

For membrane potential experiments using flow cytometry, mESCs were loaded with 1 µM bis-(1,3-dibutylbarbituric acid) trimethine oxonol (DiBAC_4_(3), Invitrogen). Cell acquisition, analysis and quantification were performed with a FACScan instrument using CellQuest Pro software (Becton Dickinson).

### Flow cytometry

ES spheres cells were dissociated using TrypLE Express (Invitrogen) to single cells, plated and cultured for 6h with clofilium (10 µM) or doxorubicin (1 µM). Doxorubicin was used as positive control. Cells were then fixed overnight in 75% ethanol and rehydrated in PBS. For staining with propidium iodide and γH2AX (1:4000, Abcam) antibody, cells were incubated with primary antibody for 1h at RT, followed by incubation with secondary antibody (1:400, Alexa Fluor 488nm, Invitrogen) for 40 min at RT. Flow cytometry was performed on a FACScan instrument using CellQuest Pro software. For live cell Erg staining, mESCs were incubated with primary antibody (1:100 dilution, Almone Lab) for 50 min at RT, followed by incubation with secondary antibody (1:1000, Alexa Fluor 488nm, Invitrogen) for 30 min at RT. For DAPI staining, cells were incubated with DAPI (1:500, 0.2mg/ml stock) for 15 min at 37° C and acquired with CyAN ADP (Dako Cytomation) and analyzed with FlowJo software (Tree Star, Ashland, OR, USA).

### Measurements of relative mass redistribution

An EPIC (Corning) instrument, a resonant wave guide grating biosensor, was used to monitor relative mass redistribution (RMD). Morphological changes were analyzed in a cell population in real-time. Prior to measurements, mESCs were attached on a laminin coated assay surface in a 96-well plate format using mild centrifugation followed by incubation at 37° C for 1 h. After initial recording of a stable baseline, compound was added and measurements continued for up to 2 h.

### Time-lapse microscopy and image analysis

mESCs were plated, as single cells or spheres, on a 12-well plate and treated with various Erg inhibitors. Time-lapse was run using a Zeiss Cell Observer microscope, acquiring one picture every 5 min for 24 h, at a 20X magnification with Zeiss AxioVision software. ImageJ was used for image analysis.

### LifeAct transfection and TIRF imaging

mESCs were plated on matrigel-coated 8 well chambers plates. Lipofectamine 2000® was used as a transfection agent and mESCs were transfected with only OptiMEM for 5 hours then the media was changed. Images were acquired on an iMIC stand (Till Photonics) with an Olympus ×100 1.45NA objective. A DPSS laser (75 mW) at 488 nm (Coherent Sapphire) was selected through an acousto-optical tunable filter. A two-axis scan head was used to adjust laser incidence angles. Images were collected with an Andor iXON DU-897 EMCCD camera controlled by the Live Acquisition (Till Photonics) software.

### Atomic force microscopy

AFM elasticity measurements were carried out using a CellHesion 200 AFM (JPK Instruments) mounted on a Zeiss Axiovert microscope. The optical microscope was used to select cells and position the cantilever tip. Additionally, brightfield images were acquired for every analyzed cell. We used triangular MLCT Microlever Probes (Veeco Probes) with a spring constant of 0.03 N/m, which were calibrated using a thermal noise method provided by the JPK CellHesion 200 control software V.3.3. Force-distance curves were acquired using a scan rate of 5 µm/s and setpoint 0.4 nN and the force curves were analysed using JPK Image Processing software. Based on the brightfield images, only round cells were analyzed. The Young’s Modulus was calculated from an average of five force-distance curves per cell. AFM measurements were performed in Leibovitz L-15 Medium at 37° C. In box and whisker charts, the ends of the whisker were set at 1.5 x IQR above the third quartile (Q3) and 1.5 x IQR below the first quartile (Q1). The maximum value outside this range was indicated.

## Supporting Information

Figure S1 Cell morphology and blebbing is altered during mitosis in ESCs. a) Time lapse imaging series of ESCs prior to during and after mitosis showing rounding up and loss of blebbing in mitosis. b) Confocal fluorescent microscopy imaging of an ESC colony with z-stack cross-sections: b’ y-z plane and b″ y-z plane. Indicated are two cells, 1 and 2, in cytokinesis rounding up and decreased surface contact with the colony. Indicated is also an interphase cell, 3, with a flatter cell morphology and closer cell–cell contact. White color indicates DAPI and green integrin α6, red line indicates cell surface of the two cells in cytokinesis.
(TIF)

Figure S2 Absolute K^+^ channel expression over the cell cycle. a) K^+^ channel transcriptome analysis by RNA sequencing of cell cycle sorted mESCs using flow cytometry and DNA staining. Expression is normalized as RPMB.
(TIF)

Figure S3 Flow cytometry plot of live mESCs stained with DAPI and plotted against forward scattering (FSC) reflecting cell volume growth from G1 to G2. (TIF)

Figure S4 a) mESCs were exposed to clofilium (10 µM) or vehicle for 6h and analyzed by immunostaining against γH2AX shown in histogram where three apparent discrete populations are indicated by roman numerals (I low γH2AX, II medium γH2AX and III high γH2AX content populations) and b) plotted against DNA content analyzed by propidium iodide labeling where a cell population with a sub-2N DNA content is indicated by a red circle.
(TIF)
