# Correction: Erg Channel Is Critical in Controlling Cell Volume during Cell Cycle in Embryonic Stem Cells

**DOI:** 10.1371/journal.pone.0090271

**Published:** 2014-03-20

**Authors:** The PLOS ONE Staff

There is an error in Figure 4. Figures 4B and 4C should have the y-axis labeled "Young's Modulus (Pa)." Please see the corrected version of Figure 4 here.

**Figure 4:**
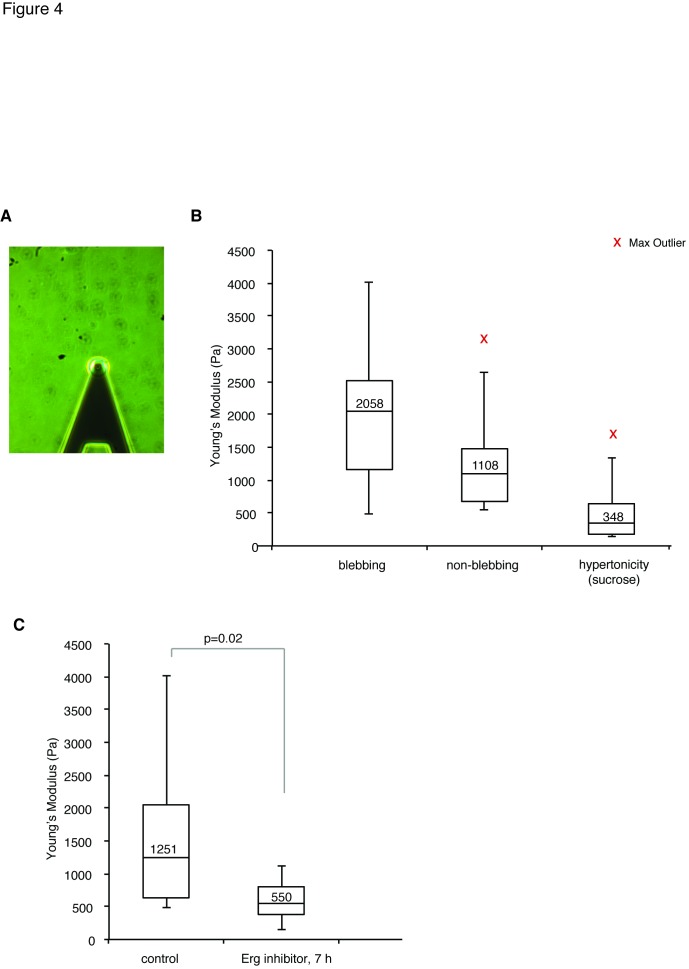
Erg inhibition decreased stiffness in mESCs. (A) A representative image of the cantilever placed over a mESC during atomic force microscopy. (B) In control conditions, blebbing cells showed a trend towards higher stiffness than non-blebbing cells (n = 7, p = 0.17, median indicated in box) and control cells that were subjected for hypertonic medium (sucrose 20 mM) for one hour showed reduced stiffness (n = 11, p<0.001). (C) After 7 h of Erg inhibition (E4031; 10 μM) treated cells (n = 8) were significantly (p = 0.02, t-test unequal variance) less stiff than control cells (n = 18).
