# Supplementary figures and images for: Erg Channel Is Critical in Controlling Cell Volume during Cell Cycle in Embryonic Stem Cells
Source: PLoS One. 2013 Aug 2;8(8):e72409. doi: 10.1371/journal.pone.0072409 (PMC3732234; doi:10.1371/journal.pone.0072409)

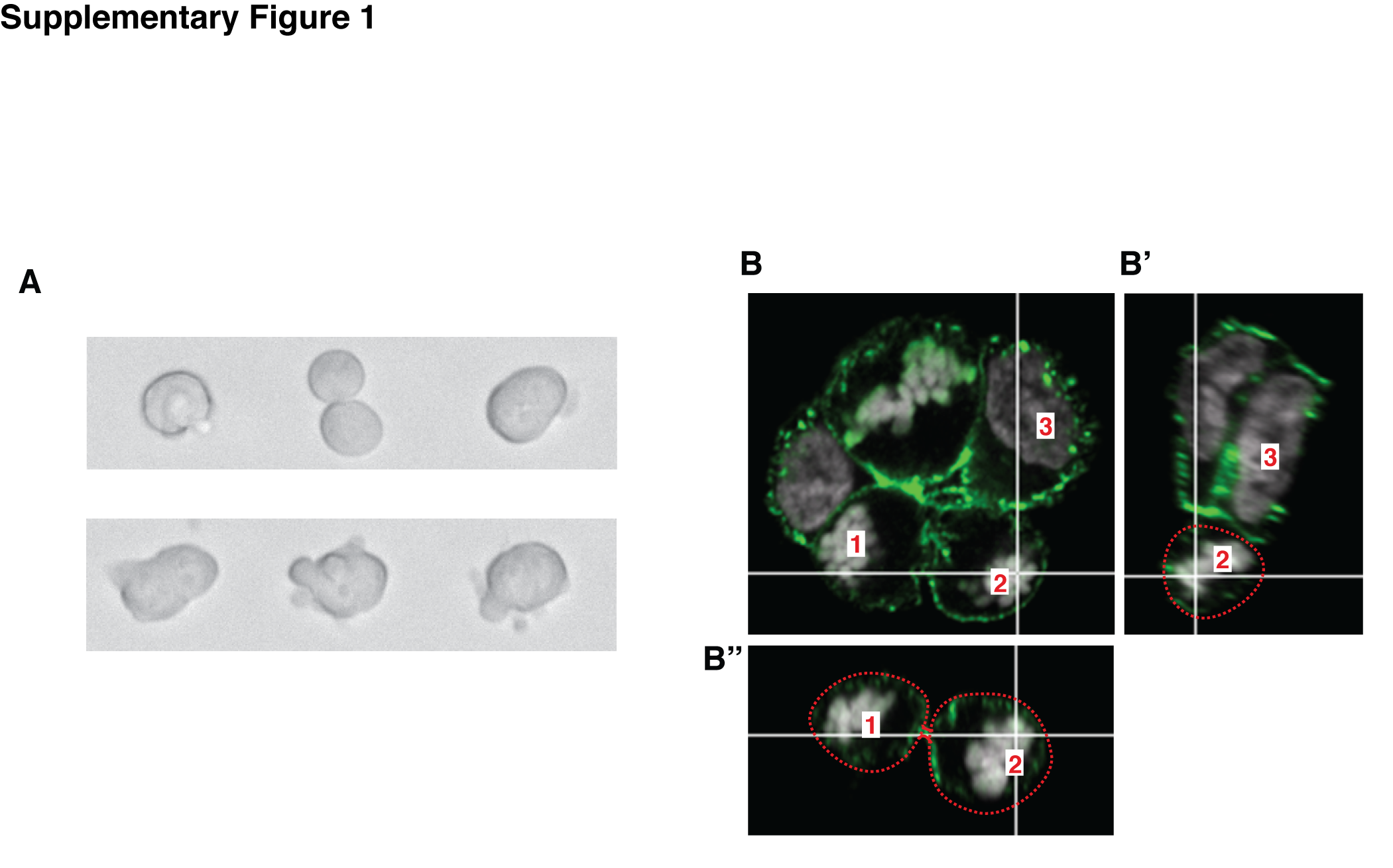

Supplement: Figure S1 — a) Time lapse imaging series of ESCs prior to during and after mitosis showing rounding up and loss of blebbing in mitosis. b) Confocal fluorescent microscopy imaging of an ESC colony with z-stack cross-sections: b’ y-z plane and b″ y-z plane. Indicated are two cells, 1 and 2, in cytokinesis rounding up and decreased surface contact with the colony. Indicated is also an interphase cell, 3, with a flatter cell morphology and closer cell–cell contact. White color indicates DAPI and green integrin α6, red line indicates cell surface of the two cells in cytokinesis. (TIF) [file pone.0072409.s001.tif]

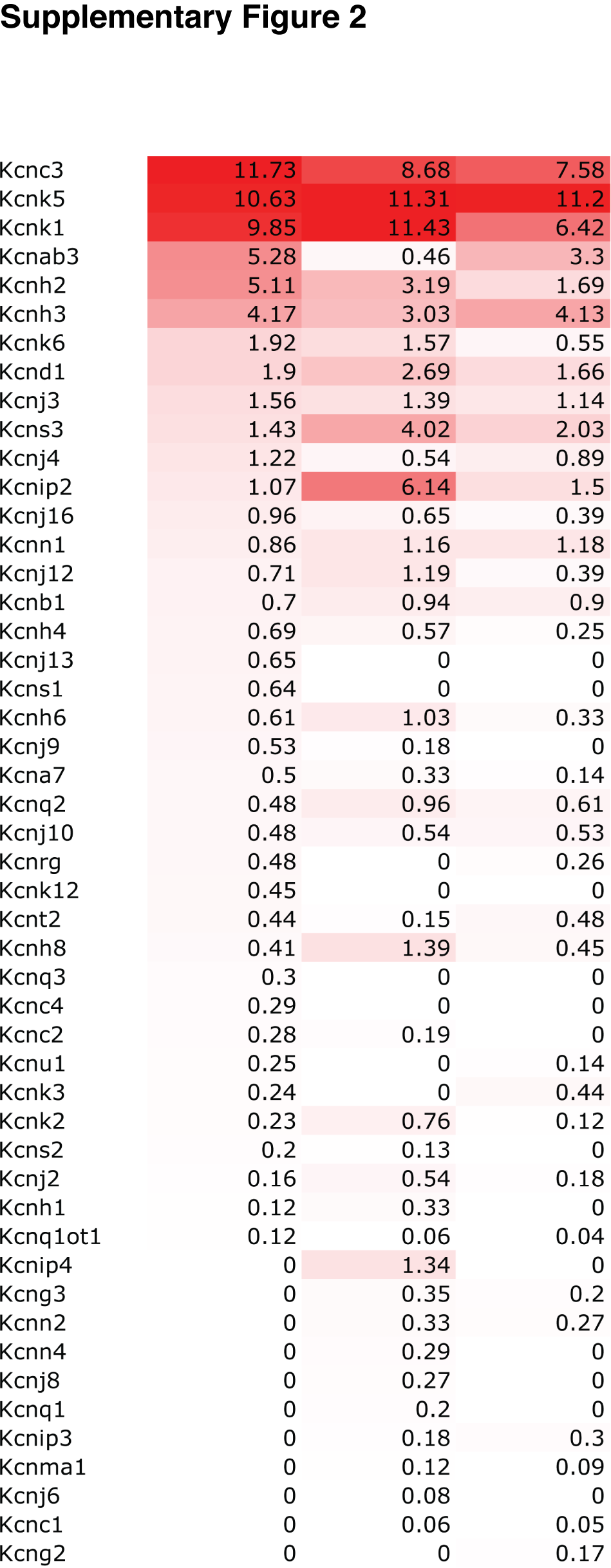

Supplement: Figure S2 — a) K+ channel transcriptome analysis by RNA sequencing of cell cycle sorted mESCs using flow cytometry and DNA staining. Expression is normalized as RPMB. (TIF) [file pone.0072409.s002.tif]

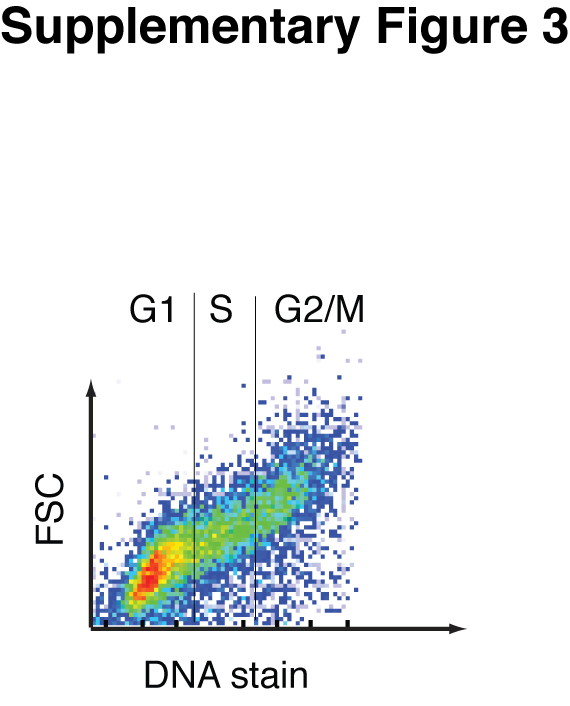

Supplement: Figure S3 — (TIF) [file pone.0072409.s003.tif]

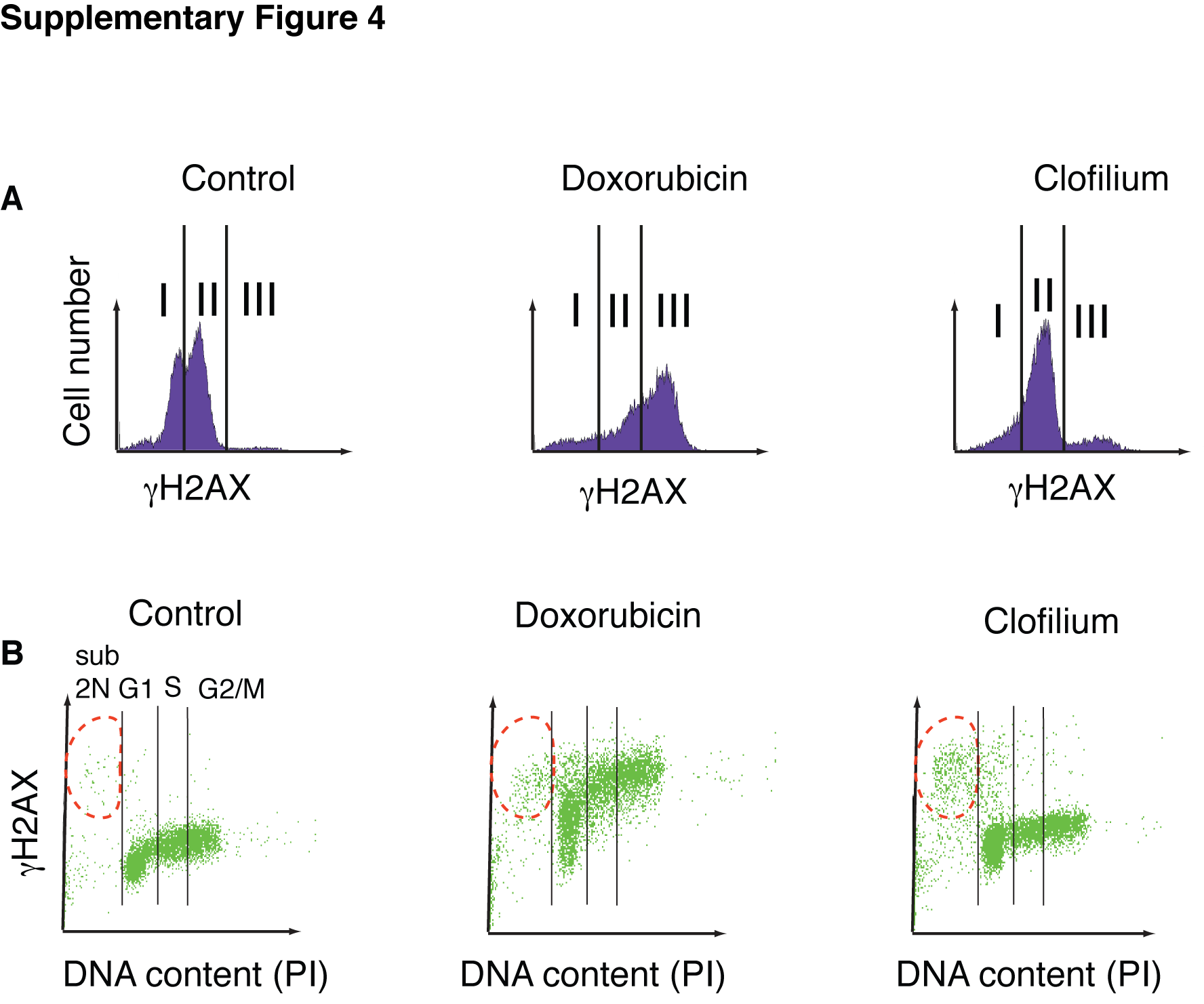

Supplement: Figure S4 — a) mESCs were exposed to clofilium (10 µM) or vehicle for 6h and analyzed by immunostaining against γH2AX shown in histogram where three apparent discrete populations are indicated by roman numerals (I low γH2AX, II medium γH2AX and III high γH2AX content populations) and b) plotted against DNA content analyzed by propidium iodide labeling where a cell population with a sub-2N DNA content is indicated by a red circle. (TIF) [file pone.0072409.s004.tif]
